# Inhibition of glycogen synthase kinase 3*β* promotes autophagy to protect mice from acute liver failure mediated by peroxisome proliferator-activated receptor *α*

**DOI:** 10.1038/cddis.2016.56

**Published:** 2016-03-24

**Authors:** F Ren, L Zhang, X Zhang, H Shi, T Wen, L Bai, S Zheng, Y Chen, D Chen, L Li, Z Duan

**Affiliations:** 1 Beijing Artificial Liver Treatment & Training Center, Beijing YouAn Hospital, Capital Medical University, Beijing, China; 2 Department of Infectious Diseases, The Third Affiliated Hospital of Hebei Medical University, Shijiazhuang, China; 3 Department of Cell Biology, Municipal Laboratory for Liver Protection and Regulation of Regeneration, Capital Medical University, Beijing, China

## Abstract

Our previous studies have demonstrated that inhibition of glycogen synthase kinase 3*β* (GSK3*β*) activity protects mice from acute liver failure (ALF), whereas its protective and regulatory mechanism remains elusive. Autophagy is a recently recognized rudimentary cellular response to inflammation and injury. The aim of the present study was to test the hypothesis that inhibition of GSK3*β* mediates autophagy to inhibit liver inflammation and protect against ALF. In ALF mice model induced by d-galactosamine (d-GalN) and lipopolysaccharide (LPS), autophagy was repressed compared with normal control, and d-GalN/LPS can directly induce autophagic flux in the progression of ALF mice. Autophagy activation by rapamycin protected against liver injury and its inhibition by 3-methyladenine (3-MA) or autophagy gene 7 (Atg7) small interfering RNA (siRNA) exacerbated liver injury. The protective effect of GSK3*β* inhibition on ALF mice model depending on the induction of autophagy, because that inhibition of GSK3*β* promoted autophagy *in vitro* and *in vivo*, and inhibition of autophagy reversed liver protection and inflammation of GSK3*β* inhibition. Furthermore, inhibition of GSK3*β* increased the expression of peroxisome proliferator-activated receptor *α* (PPAR*α*), and the downregulated PPAR*α* by siRNA decreased autophagy induced by GSK3*β* inhibition. More importantly, the expressions of autophagy-related gene and PPAR*α* are significantly downregulated and the activity of GSK3*β* is significantly upregulated in liver of ALF patients with hepatitis B virus. Thus, we have demonstrated the new pathological mechanism of ALF that the increased GSK3*β* activity suppresses autophagy to promote the occurrence and development of ALF by inhibiting PPAR*α* pathway.

Acute liver failure (ALF), an inflammation-mediated hepatocellular injury process, is a clinical syndrome that results from hepatocellular apoptosis and hemorrhagic necrosis.^1^ ALF frequently results from viral hepatitis, ingestion of drugs or toxic substances, or hepatic ischemia-reperfusion injury, among others. The prognosis for ALF is extremely poor, and there is currently no effective therapy for the end stage of the disease other than liver transplantation.^2^ Although the nature of ALF has been extensively studied, the mechanisms by which organ damage occurs are not completely understood.

Glycogen synthase kinases 3 are a group of ubiquitously expressed serine/threonine kinases that are initially found to regulate glycogen synthesis. There are two highly homologous isoforms, designated as glycogen synthase kinase 3 (GSK3)*α* and GSK3*β*, respectively. Constitutively active in resting cells, GSK3*β* has a broad range of substrates, and regulates cell activation, differentiation and survival.^3, 4^ Among the diverse functions that are regulated by GSK3*β*, inflammation has recently emerged as one of the major interesting focuses. Studies showed that GSK3*β* is an important positive regulator in inflammatory process.^5, 6, 7, 8^ GSK3*β* deletion results in embryonic lethality caused by severe liver degeneration during development.^9^ Particularly, GSK3*β*-deficient cells become more sensitive to tumor necrosis factor *α* (TNF-*α*)-induced apoptosis.^10^ Our studies have shown that the activity of GSK3*β* is promoted in the progression of ALF and inhibition of GSK3*β* mitigates liver inflammation to ameliorate ALF model of mice,^11, 12^ but its protective mechanisms are not well defined.

Macroautophagy (referred to hereafter as autophagy) is a highly evolutionarily conserved process found in virtually all types of eukaryotic cells. Autophagy involves the sequestration of regions of cytosol within double-membrane-bound compartments followed by lysosome-based degradation of the contents. Previous studies have suggested that autophagy represents an adaptive strategy by which cells can remove damaged organelles and enhance survival following bioenergetics-induced stress, and have multiple roles of autophagy in the regulation of cell death, differentiation and the anti-microbial response in mammals.^13, 14, 15^ In recent years, emerging evidence has indicated that the autophagy process may have an essential role for the host during bacterial clearance and may also interact with inflammatory processes, which consequently may impact the outcomes of disease progression.^16, 17^ There is a complex reciprocal relationship between autophagy pathway/proteins and inflammation.^18, 19^ Recent observations have revealed a relationship between autophagy and inflammasome-associated pro-inflammatory cytokine maturation in macrophages.^20, 21^

Given the above information, we speculated that autophagy activation may serve a protective function to restrain liver inflammation in cases of ALF. The study has also showed that inhibition of hepatocyte autophagy increases TNF*α*-dependent liver injury by promoting caspase-8 activation.^22^ So, we hypothesized that inhibition of GSK3*β* may promote autophagy to protect mice from ALF. To test these hypotheses, we used the ALF model induced by the co-injection of d-galactosamine (d-GalN) and lipopolysaccharide (LPS), which has been widely used to examine the underlying mechanisms of ALF,^23, 24^ to explore the protective mechanisms of GSK3*β* inhibition and its regulatory pathway in the context of ALF, and further measure the expression of autophagic gene in human liver samples from patients with ALF. Our findings demonstrate that inhibition of GSK3*β* increase autophagy to alleviate liver inflammation and protect mice from ALF mediated by peroxisome proliferator-activated receptor *α* (PPAR*α*).

## Results

### Dynamic profile of autophagy in the progression of d-GalN/LPS-induced ALF

**Figure 1:**
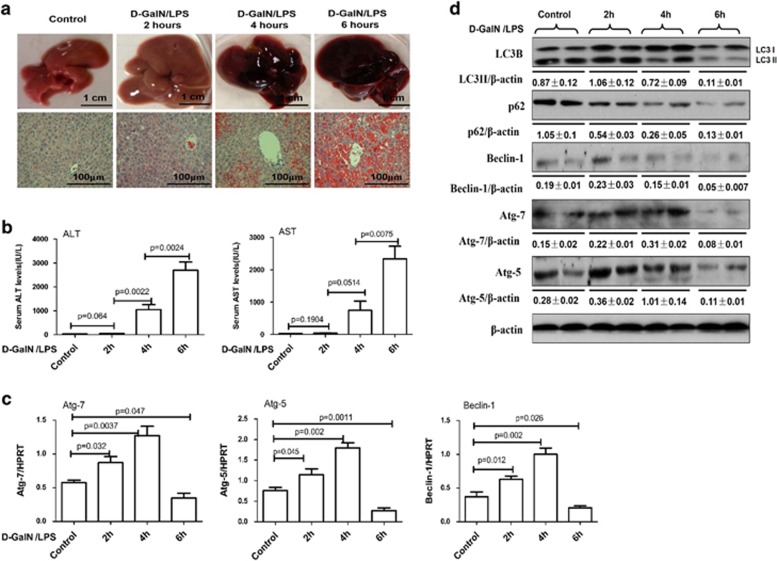
The regulation of autophagy during d-GalN/LPS induced mice ALF progression. Mice were intraperitoneally injected with d-GalN (700 mg/kg) and LPS (10 *μ*g/kg) at 2, 4 and 6 h (12 mice per group). The mice in the control group (*n*=8) were injected with PBS only. (**a**). Representative livers and H&E staining of livers. (**b**) Serum AST and ALT enzyme levels. (**c**) Gene expressions of Beclin-1, Atg5 and Atg7 were measured by qRT-PCR in the livers. (**d**) Protein expression levels of LC3B, p62, Beclin-1, Atg5 and Atg7 were measured by western blot assays in the livers. A representative blot from two samples of every group is shown

We first investigated a possible association between autophagy and ALF induced by d-GalN/LPS treatment. We found that massive hepatic injury was apparent after 4–6 h as revealed by gross morphology of the liver; and the liver tissue of mice appears spotty hemorrhage at 2 h; the increased inflammation, hepatic lobules disorder, a large number of inflammatory cell infiltration and visible hepatocyte apoptosis or confluent necrosis are shown at 4 h; there are a lot of visible large necrosis areas in the liver tissue, the entire liver congestion at 6 h (Figure 1a). The results of gross morphology of the liver and hematoxylin and eosin (H&E) staining were in agreement with the increased serum alanine aminotransferase (ALT) and aspartate aminotransferase (AST) enzyme levels (Figure 1b). Therefore, a mouse model of ALF was successfully constructed at 6 h time point after the d-GalN/LPS treatment, which was employed for all subsequent experiments. Accompanying the liver injury, compared with normal mice, the mRNA or protein levels of autophagic gene including Atg5, Atg7, Beclin-1 and LC3II conversion were gradually upregulated after 2 h, but declined after 6 h (Figures 1c and d; Supplementary Figure 1). The p62 protein, also called sequestosome 1 (SQSTM1), binds directly to LC3 and GABARAP family proteins via a specific sequence motif. The protein is itself degraded by autophagy and may serve to link ubiquitinated proteins to the autophagic machinery to enable their degradation in the lysosome; as p62 accumulates when autophagy is inhibited, and decreased levels can be observed when autophagy is induced, p62 may be used as a marker to study autophagic flux.^25^ As showed in Figure 1d, p62 protein degradation is constantly increased in progression of d-GalN/LPS-induced liver injury. Taken together, these results indicated that autophagy is repressed in the mice ALF model.

### Enhanced autophagy ameliorates hepatotoxicity induced by d-GalN/LPS

**Figure 2:**
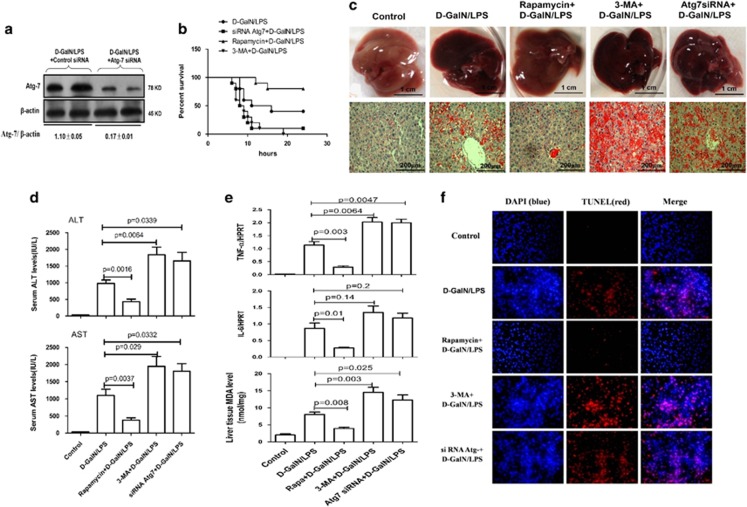
Autophagy protects against d-GalN/LPS-induced liver injury rapamycin+d-GalN/LPS-treated mice were administered rapamycin (2 mg/kg, i.p.) 2 h before d-GalN/LPS exposure (*n*=12); 3-MA+d-GalN/LPS-treated mice were administered 3-MA (10 mg/kg, i.p.) 2 h before d-GalN/LPS exposure (*n*=12); siRNA Atg7+d-GalN/LPS-treated mice were pretreated with siRNA Atg7 (3 mg/kg, i.v.) for 48 h before d-GalN/LPS exposure (*n*=12). Control mice were pretreated with vehicle (DMSO) 2 h before PBS injection (*n*=8). (**a**) Protein expression levels of Atg7 and *β*-actin were measured by western blotting. A representative blot for two samples from every group is shown. (**b**) The survival rate of mice was measured in the Atg7 siRNA +d-GalN/LPS-, rapamycin+d-GalN/LPS- and 3-MA+d-GalN/LPS-treated group and the d-GalN/LPS-treated group (10 mice per group). (**c**) Representative livers and H&E staining of livers from different groups. (**d**) Serum AST and ALT enzyme levels from different groups. (**e**) Gene expression levels of TNF-*α* and IL-6 were measured by qRT-PCR and the level of MDA was measured in livers from different groups. (**f**) TUNEL staining (red) liver tissue at 6 h after d-GalN/LPS administration. Representative of one experiment is shown. Original magnification × 200

We then evaluated the physiological roles of autophagy in d-GalN/LPS-induced ALF. Pretreatment with rapamycin for 2 h before d-GalN/LPS injection protected mice against ALF; and pretreatment with 3-methyladenine (3-MA) for 2 h or Atg7 siRNA for 24 h before d-GalN/LPS injection further aggravated the liver injury of ALF. The inhibition of liver Atg7-specific siRNA *in vivo* was confirmed by the reduced Atg7 protein levels in the mice (Figure 2a). In the survival analysis, the mice in the d-GalN/LPS control group began to perish 7 h after d-GalN/LPS injection, and the survival rate of these mice was 40% (4 of 10 mice) at the 24-h time point. By contrast, the survival rate after rapamycin treatment was 80% (8 of 10 mice); the survival rate after 3-MA or Atg7 siRNA treatment was 0% (0 of 10 mice) and 10% (1 of 10 mice), respectively (Figure 2b). The gross morphology of the liver after rapamycin treatment appeared substantially normal, and the liver architecture was well preserved, such as decreased inflammatory cell infiltration and less hepatocyte apoptosis or confluent necrosis; but pretreatment of d-GalN/LPS-induced mice with 3-MA or Atg7 siRNA results in more massive hepatic toxicity as revealed by gross morphology of the liver and the liver architecture (Figure 2c). Compared with the ALF group, the mice subjected to rapamycin treatment showed significantly lower sALT and sAST levels, the genes expression level of TNF-*α* and interleukin (IL)-6, and the level of malondialdehyde (MDA) in liver; but 3-MA or Atg7 siRNA pretreatment further aggravated liver functions, and significantly increased the level of TNF-*α* and MDA (Figures 2d and e); Moreover, by TUNEL (terminal deoxynucleotidyl transferasemediated dUTP nick-end labeling) assay, pretreatment with rapamycin significantly decreased hepatocyte apoptosis and 3-MA or Atg7 siRNA increased the hepatocyte apoptosis (Figure 2f). These results demonstrated that autophagy is critical for d-GalN/LPS-induced ALF and that its induction protects mice from liver injury.

### Inhibition of GSK3*β* promotes autophagy induced by starvation *in vitro*

**Figure 3:**
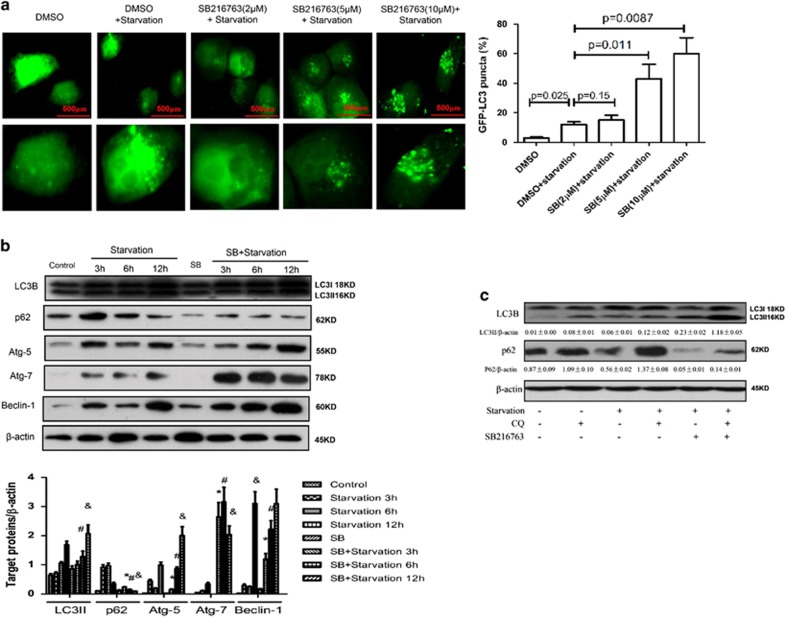
Inhibition of GSK3*β* further promotes autophagy induced by starvation *in vitro.* (**a**). Transfected GFP-LC3 plasmid for 12 h, the primary hepatocytes were pre-incubated with SB216763 (2, 5 or 10 *μ*M) for 12 h to observe the formation of autophagosomes. (**b**) The primary hepatocytes were stimulated with or without SB216763 for 30 min, followed for 3, 6 or 12 h. The cell lysates were analyzed by western blot in different time. The levels of LC3B, p62, Atg7, Atg5, Beclin-1and *β*-actin were measured by western blotting. The graph of densitometry analysis should be shown (compared with starvation 3 h group: *P<0.05; compared with starvation 6 h group: ^#^*P*<0.05; compared with starvation 12 h group: ^&^*P*<0.05). (**c**) Inhibition of GSK3*β* induced autophagic flux in primary hepatocytes under starvation. Western blot analysis of expression of the LC3B and p62. A representative results from three independent experiments is shown. Densitometry analysis of the proteins was performed for each sample

Next, we investigated the regulation of autophagy by the effects of GSK3*β* inhibition on primary hepatocyte in response to starvation. We transfected the GFP-LC3 plasmid into hepatocyte to observe the formation of autophagosomes. As shown in Figure 3a, the GFP-LC3 signal was weak in the control cells, but was bright and punctate after SB216763 treatment, which is a specific inhibitor of GSK3*β* activity, in a dose-dependent manner. Western blot results showed that SB216763 treatment promoted the expression level of LC3II, Atg5, Beclin-1 and Atg7 proteins; meanwhile, SB216763 treatment also increased the degradation of p62 (Figure 3b). Furthermore, autophagic flux in hepatocyte was monitored after treatment with SB216763 in the presence or absence of chloroquine (CQ). The addition of CQ further increased LC3II expression, and decreased the degradation of p62 compared with cells treated with SB216763 and starvation (Figure 3c). Taken together, the results showed that inhibition of GSK3*β* promotes autophagic flux in hepatocyte treated by starvation *in vitro*.

### Inhibition of GSK3*β* promotes autophagy in liver of ALF mice induced by d-GalN/LPS

**Figure 4:**
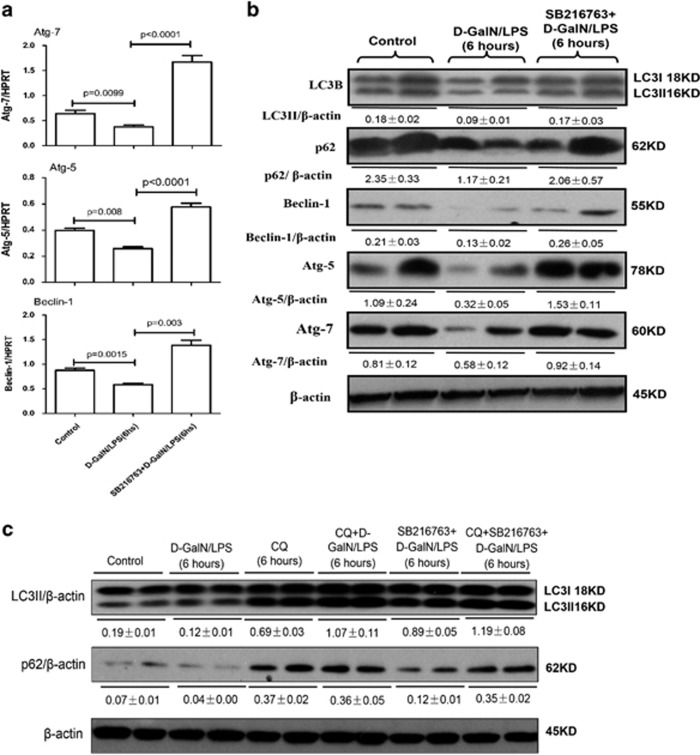
Autophagy is promoted by GSK3*β* signaling molecule *in vivo*. The mice were pretreated with or without SB216763 (25 mg/kg, i.p.) for 2 h, followed by d-GalN/LPS for 6 h stimulation (*n*=10). CQ-treated mice were pretreated with CQ (60 mg/kg, i.p.) for 8 h (*n*=10); CQ+ d-GalN/LPS-treated mice were pretreated with CQ (60 mg/kg, i.p.) for 2 h before d-GalN/LPS exposure (*n*=10); CQ+SB216763+d-GalN/LPS-treated mice were pretreated with CQ (60 mg/kg, i.p.) and SB216763 for 2 h before d-GalN/LPS exposure (*n*=10). (**a**) Gene expression levels of autophagy-related proteins, including Atg7, Atg5 and Beclin-1 were measured by qRT-PCR in livers of control mice group, d-GalN/LPS-induced ALF mice group and SB216763 pretreament ALF group induced by d-GalN/LPS. (**b**) Protein expression levels of autophagy-related proteins, including LC3B, p62, Atg7, Atg5 and Beclin-1, were measured by western blotting in livers. Two representative blot for liver samples from each group were shown. (**c**) Protein expression levels of LC3B and p62 were measured by western blotting in livers. A representative blot for three samples from each group is shown

To further confirm our *in vitro* experimental findings, we investigated the regulation of autophagy by the effects of GSK3*β* inhibition on ALF mice induced by d-GalN/LPS. qRT-PCR (quantitative reverse transcription PCR) results showed that, compared with d-GalN/LPS-treated mice, GSK3*β* inhibition by SB216763 pretreatment in ALF mice promoted the expression of Atg7, Atg5 and Beclin-1 genes, these alterations were confirmed by western blot analyses; interestingly, GSK3*β* inhibition in ALF mice also promoted LC3II conversion and decreased p62 protein degradation in d-GalN/LPS-induced mice (Figures 4a and b). The CQ pretreatment further increased LC3II conversion and decreased p62 degradation compared with mice treated with SB216763 and d-GalN/LPS. Taken together, inhibition of GSK3*β* promotes autophagosomes and blocks autophagolysosome formation in d-GalN/LPS-induced ALF mice.

### Inhibition of GSK3*β* protects mice from ALF through autophagy-mediated inflammatory response

**Figure 5:**
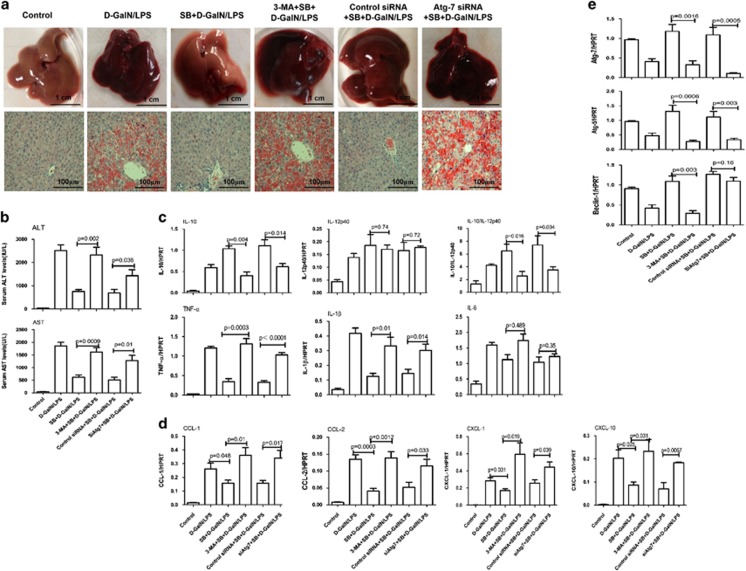
The inhibition of GSK3*β* activity protects mice from ALF through autophagy mechanisms. The mice were pretreated with or without SB216763 (25 mg/kg, i.p.) for 2 h, followed by d-GalN/LPS for 6 h stimulation (*n*=10). SB216763+3-MA+ d-GalN/LPS-treated mice were co-administered 3-MA (10 mg/kg) and SB216763 at 2 h before d-GalN/LPS exposure (*n*=10); SB216763+siAtg7+ d-GalN/LPS-treated mice were pretreated with Atg7 siRNA (3 mg/kg) for 48 h via tail vein injection and then administered SB216763 2 h before d-GalN/LPS exposure (*n*=11). (**a**) Representative livers and H&E staining of livers (200 ×) from different groups. (**b**) Serum AST and ALT enzyme levels from different groups. (**c**) Gene expression of cytokines including TNF-*α*, IL-1*β* and IL-6 at 6 h, IL-12p40 and IL-10 at 2 h after d-GalN/LPS injection. (**d**) Gene expression of chemokines including CCL-1, CCL-2, CXCL-1 and CXCL-10 at 6 h after d-GalN/LPS injection. (**e**) Gene expression of Atg7, Atg5 and Beclin-1 at 6 h after d-GalN/LPS injection

Next, we sought to explore whether GSK3*β* inhibition leads to the induction of autophagy to protect the liver from injury. The results showed that hepatic protection by SB216763 in ALF was partially negated by 3-MA or siRNA Atg7, which was evident by the relatively worse gross morphology, and the relatively less preserved liver architecture by histology (Figure 5a), and the significantly higher sALT and sAST levels (Figure 5b). Thus, these results demonstrated that hepatoprotective mechanisms of GSK3*β* inhibition depend on autophagy pathways.

Given the ability of autophagy to elaborately regulate inflammation and GSK3*β* inhibition is also able to control liver inflammation in d-GalN/LPS-induced ALF, we sought to determine whether an autophagy pathway is required for GSK3*β* inhibition-mediated suppression of the inflammatory response. In the context of GSK3*β* inhibition in the ALF mice, 3-MA or siRNA Atg7 restored the gene expression of TNF-*α*, IL-1*β*, IL-6 and IL-12p40 (Figure 5c). Furthermore, autophagy inhibition upregulated the gene expression of chemokines again (Figure 5d). Meanwhile, compared with SB216763-pretreated ALF mice, 3-MA further reversed the genes expression of Atg7, Atg5 and Beclin-1, although Atg7 siRNA only reversed the genes expression of Atg5 (Figure 5e). These results demonstrated that the autophagy pathways induced by GSK3*β* inhibition may contribute to the suppression of liver inflammation in ALF.

### Inhibition of GSK3*β* mediated promoting PPAR*α* induces autophagy pathway

**Figure 6:**
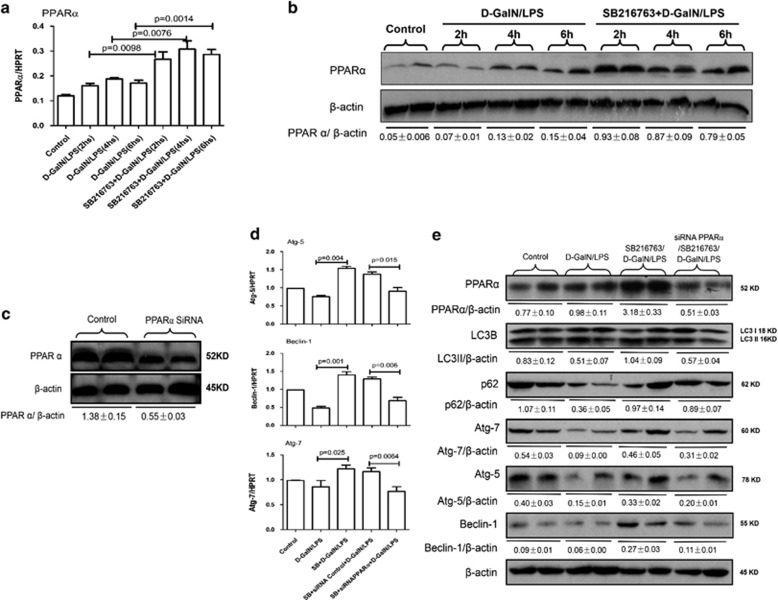
Autophagy is regulated by GSK3*β*-PPAR*α* signaling pathway in ALF. (**a** and **b**) The mice were pretreated with or without SB216763 (25 mg/kg, i.p.) for 2 h, followed by d-GalN/LPS for 2, 4 or 6 h stimulation(*n*=10 mice per every group). Gene and protein expression levels of PPAR*α* at 2, 4 and 6 h after d-GalN/LPS injection in livers from every groups. A representative blot for two samples from each group is shown. (**c**) SiRNA control- or siRNA PPAR*α*-treated mice were injected with SiRNA control or PPAR*α* siRNA (3 mg/kg) for 48 h via tail vein injection (*n*=10); Mice were sacrificed 48 h after siRNA PPAR*α* treatment. Protein expression level of PPAR*α* was measured by western blotting in livers. A representative blot for two samples from each group is shown. (**d** and **e**) SB+siRNA PPAR*α*+d-GalN/LPS-treated mice were pretreated with PPAR*α* siRNA (3 mg/kg) for 48 h via tail vein injection and then administered SB216763 2 h before d-GalN/LPS exposure (*n*=12); SB+siRNA control+d-GalN/LPS-treated mice were pretreated with control siRNA (3 mg/kg) for 48 h via tail vein injection, then administered SB216763 2 h before d-GalN/LPS exposure (*n*=10). Gene expression levels of autophagy-related proteins were measured by qRT-PCR in livers. Protein expression levels of PPAR*α* and autophagy-related proteins were measured by western blotting in livers. Two representative blot for liver samples from each group were shown

Because our previous study has shown that PPAR*α* activation promotes autophagy to suppress the inflammatory response in ALF,^26^ we evaluated whether GSK3*β* inhibition promotes autophagy via a PPAR*α*-dependent pathway in the context of ALF. qRT-PCR and western blotting results showed that, compared with d-GalN/LPS-treated mice, GSK3*β* inhibition by SB216763 pretreatment in ALF mice promoted the expression of PPAR*α* gene and protein (Figures 6a and b). Next, we sought to confirm that GSK3*β* inhibition leads to the induction of autophagy by PPAR*α* activation. We applied siRNA to knockdown PPAR*α* and the inhibition of liver PPAR*α*-specific siRNA *in vivo* was confirmed by the reduced PPAR*α* levels in the mice (Figure 6c). Compared with SB216763-pretreated ALF mice induced by d-GalN/LPS, the PPAR*α* siRNA pretreatment reversed the gene and protein expression of Atg7, Atg5 and Beclin-1 induced by SB216763; importantly, the PPAR*α* siRNA also decreased the lipidation of LC3I to LC3II and mildly increased the degradation of p62 (Figures 6d and e). Moreover, we further explored whether GSK3*β* inhibition protects liver from injury through the induction of PPAR*α* in ALF mice. The results showed that hepatic protection by SB216763 in ALF was partially reversed by PPAR*α* siRNA, which was evident by the relatively worse gross morphology, and the relatively less preserved liver architecture by histology (Supplementary Figure 2A), and the significantly higher sALT and sAST levels compared with SB216763-pretreated ALF mice group (Supplementary Figure 2B). Furthermore, in the context of GSK3*β* inhibition in the ALF mice, PPAR*α* siRNA restored the gene expression of pro-inflammatory cytokines, such as TNF-*α*, IL-1*β*, IL-12p40 and chemokines including CCL-1 (chemokine (C-C motif) ligand), CCL-2, CXCL-1 (chemokine (C-X-C motif) ligand) and CXCL-10 (Supplementary Figure 2C). Thus, these results demonstrated that inhibition of GSK3*β* induces autophagy pathway in a PPAR*α*-dependent manner in ALF.

### Decreased expression of autophagy in the liver of ALF patients with HBV infection

**Figure 7:**
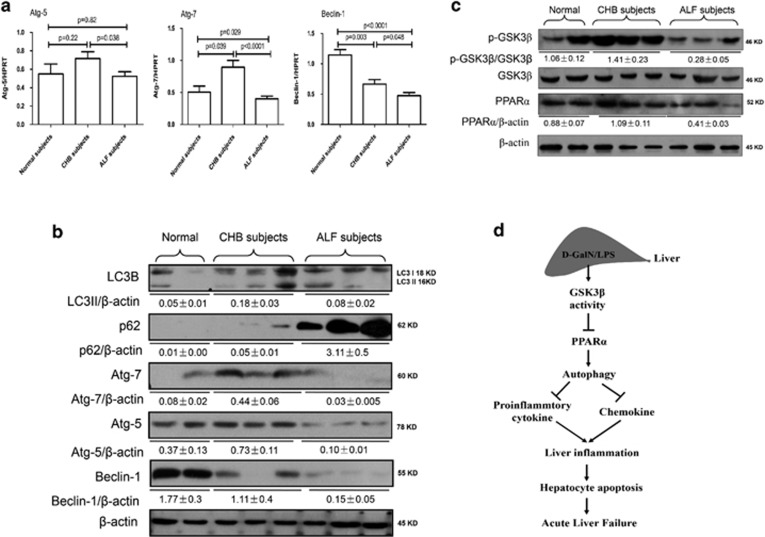
Suppression of autophagy in the liver of ALF patients with HBV infection. (**a**) Gene expression levels of Atg7, Atg5 and Beclin-1 were measured by qRT-PCR in the livers of normal subjects (*n*=9), CHB patients (*n*=14) and ALF patients (*n*=19). The average target gene/HPRT ratios for each experimental group were plotted. (**b**) Protein expression levels of LC3B, p62, Atg7, Atg5 and Beclin-1 were measured by western blotting in the livers of normal subjects (*n*=9), CHB patients (*n*=14) and ALF patients (*n*=19). A representative blot from three samples of every group is shown. (**c**) Protein expression levels of p-GSK3*β*, GSK3*β* and PPAR*α* were measured by western blotting in the livers of normal subjects (*n*=9), CHB patients (*n*=14) and ALF patients (*n*=19). A representative blot from three samples of every group is shown. (**d**) In the d-GalN/LPS-induced ALF mice, d-GalN/LPS treatment induces increase of GSK3*β* activity, and then suppresses the expression level of PPAR*α*, which promotes down-regulation of autophagy and increases the expression of pro-inflammatory cytokines and chemokines. These events lead to incremental liver inflammation, and promote the hepatocyte apoptosis and ultimately induce the development of ALF

To determine whether autophagy participates in the progression of ALF in patients with hepatitis B virus (HBV) infection, we utilized liver tissues to measure the changes of autophagy among normal controls, chronic hepatitis B (CHB) patients and ALF patients with HBV infection. Relative to the normal controls, the qRT-PCR results showed that Atg7 and Atg5 gene expression levels were significantly increased in the cases of CHB, but were significantly reduced in the cases of ALF, but Beclin-1 gene expression was gradual decreased from the cases of CHB to ALF (Figure 7a); similar results were obtained by western blot analyses in liver tissue (Figure 7b); furthermore, the expression of LC3II was increased in CHB subjects and decreased in ALF subjects, and the degradation of p62 was significant decreased in the cases of ALF (Figure 7b). Moreover, relative to the normal controls, the western blotting results showed that the activity of GSK3*β* was downregulated in cases of CHB and upregulated in cases of ALF, and PPAR*α* expression levels were not different in the cases of CHB, but were significantly reduced in the cases of ALF (Figure 7c). These results indicated that autophagy is depressed in ALF subjects induced by HBV infection, and it is likely to be regulated by GSK3*β*-PPAR*α* pathway.

## Discussion

In the present study, we demonstrated that autophagy is suppressed in d-GalN/LPS-induced ALF mice and ALF patients induced by HBV infection. Moreover, GSK3*β* inhibition resulted in elevated autophagy *in vitro* and *in vivo*, and autophagy inhibition reversed the hepatoprotective effect of GSK3*β* inhibition and restored the inflammatory response in ALF mice. Additionally, GSK3*β* inhibition promoted PPAR*α* expression to increase autophagy in ALF mice. Hence, GSK3*β*-PPAR*α*-autophagy pathway is essential to the liver inflammation mechanism in the ALF immune response cascade (as depicted in Figure 7d).

Although several previous investigations have suggested that autophagy serves as an adaptive response to cellular stress that avoids cell death,^27^ whether autophagy activation exerts protective properties in ALF mice models induced by d-GalN and LPS appears contradictory conclusions^22, 28, 29^ and the role of autophagy in the context of ALF patients induced by HBV infection are still scarce. Wang *et al.*^28^ and Amir *et al.*^22^ showed that autophagy is hepatoprotective in d-GalN/LPS-induced ALF mice model; but Li *et al.*^29^ showed that pretreatment with wortmannin alleviates d-GalN/LPS-induced acute liver injury by inhibit autophagy. Our results further confirmed that autophagy is promoted in the early phase and inhibited in the latter phase of liver injury (ALF phase) induced by d-GalN/LPS, and autophagy have a protective role in d-GalN/LPS-induced ALF mice.

What is the underlying molecular mechanism mediating the effect of autophagy in d-GalN/LPS-induced mice ALF models? The autophagy regulated by GSK3*β*-PPAR*α* signaling pathway is a novel finding. Our lasted research shows that GSK3*β* has a key role in the pathogenesis of ALF, and the inhibition of GSK3*β* activity protect liver from acute injury by reducing liver inflammation.^21^ The studies have shown that GSK3*β* can regulate autophagy, but how to regulate autophagy, the different studies have given a different conclusion. The previous study suggested that lithium (an inhibitor of GSK3*β* activity) can induce autophagy by inhibiting inositol monophosphatase,^30^ however, the other study showed that lithium can reduce autophagy and apoptosis after neonatal hypoxia-ischemia.^31^ Inhibition of GSK3*β* activity using SB216763 or down-regulation of gene expression of GSK3*β* using interfering RNA, respectively, can promote autophagy to reduce cadmium-induced apoptosis;^32^ and the differential roles of GSK3*β* during myocardial ischemia and ischemia/reperfusion by regulating autophagy.^33^ In this paper, we definitely proved that GSK3*β* inhibition not only promotes autophagy but also has the differential influence on autophagolysosome formation *in vivo* and *in vitro*. In d-GalN/LPS-induced mice ALF, inhibition of GSK3*β* activity inhibits autophagolysosome formation and blocks the autophagic flux; but in starvation-treated hepatocyte with SB216763, inhibition of GSK3*β* promotes autophagic flux. The reason why such a difference may be due to the differential pathological mechanisms *in vivo* and *in vitro* experiments: for d-GalN/LPS-induced ALF mice, LPS-induced inflammation storm to cause a large number of liver cells apoptosis and necrosis; and, *in vitro*, the state of self-protection of hepatocyte is induced under starvation stress conditions. Whether or not, GSK3*β* is one of the important regulatory molecules of cell autophagy in the pathogenesis of ALF.

PPARs are members of the nuclear hormone receptor superfamily of ligand-activated transcription factors, with its subfamily consisting of three members: PPAR*α*, PPAR*β* and PPAR*γ*.^34^ PPAR*α* has been reported to be involved in a number of cellular processes, including lipid and lipoprotein metabolism, apoptosis and inflammatory responses. PPAR*α*-null mice exhibit an aggravated reaction to various inflammatory stimuli in the skin, blood vessels, intestine and lung,^35, 36, 37, 38^ and PPAR*α* exhibits potent anti-inflammatory activity through suppressing nuclear factor-*κ*B(NF-*κ*B)and/or modulating the activation (phosphorylation) of signal transducer and activator of transcription1 (STAT1)-related inflammatory signaling in cultured neuronal cells.^39, 40^ Recently, in a model of lipopolysaccharide (LPS)-induced hepatic inflammatory injury, reduced PPAR*α* expression was shown to be associated with increased tissue bacterial load in sepsis.^41^Additionally, a lack of PPAR*α* exacerbates LPS-induced liver toxicity through STAT1 inflammatory signaling and increases oxidative/nitrosative stress.^42^ Our recent study found that, in both the mouse model and human ALF subjects, PPAR*α* is significantly downregulated in the injured liver, and PPAR*α* activation attenuates the inflammatory response to protect the liver from acute failure by promoting the autophagy pathway.^26^ In this paper, we further found that the inhibition of GSK3*β* activity increases PPAR*α* expression and inhibition of PPAR*α* restored the down-regulation of the autophagy induced by GSK3*β* inhibition in the liver. We demonstrated a new pathogenic mechanism of ALF: that the increased GSK3*β* activity-mediated PPAR*α* suppress autophagy to promote the inflammatory response which induces hepatocyte apoptosis, as a result, which contributes to the development and progression of ALF.

In summary, this study adds to the general understanding of mechanisms of ALF and provides new insight into the importance of autophagy in regulating liver injury, partially regulated by a GSK3*β*-PPAR*α* pathway. Therefore, autophagy activation represents a potent strategy to ameliorate ALF pathology. Further preclinical studies on autophagy agonists are warranted for the development of a clinically applicable therapeutic strategy against ALF.

## Materials and Methods

### Animal experiments

Male wild-type (WT, C57BL/6) mice (8–12 weeks of age) were purchased from Capital Medical University (Beijing, China) and housed in the Capital Medical University animal facility under specific pathogen-free condition and received humane care according to Capital Medical University Animal Care Committee guidelines.

The mice were intraperitoneally injected with d-GalN (700 mg/kg; Sigma, St. Louis, MO, USA) and LPS (10 *μ*g/kg; InvivoGen, San Diego, CA, USA) to induce ALF or with saline in the control animals. Control mice received the same volume of saline. The specific inhibitor of GSK3*β*, SB216763 (25 mg/kg; Sigma), was given i.p. to mice 2 h before the administration of d-GalN/LPS. Autophagy is negatively regulated by mTOR, whose activity can be inhibited by rapamycin, a lipophilic macrolide antibiotic that is a well-established inducer of autophagy; 3-MA can block autophagy through its effect on class III phosphatidylinositol 3-kinase and CQ is common chemical medicine to inhibit the fuse of lysosomes and autophagosomes, which contributes to observe the autophagic flux. Therefore, for autophagy inhibition or induction, 3-MA (10 mg/kg; Sigma), CQ (60 mg/kg; Sigma) or rapamycin (2 mg/kg; Sigma) were given i.p. to mice 2 h before the administration of d-GalN/LPS. Furthermore, suppression of autophagy was also achieved by tail vein injection of siRNA for autophagy gene 7 (Atg7, 3 mg /kg). Suppression of PPAR*α* was achieved by tail vein injection of siRNA for PPAR*α* (3 mg/kg); the mice were sacrificed at various time points after d-GalN/LPS treatment, and liver and serum samples were collected for future analysis.

### Serum aminotransferase activities

Plasma samples were taken from the mice at 6 h after d-GalN/LPS injection. Serum levels of ALT, AST as markers of hepatic damage were measured by using a multiparameteric analyzer (AU 5400, Olympus, Japan), according to an automated procedure.

### Histopathological analysis

Liver tissues were fixed in formalin and embedded in paraffin wax, and sections in 5 μm were stained with H&E using a standard protocol, and then analyzed by light microscopy.

### Quantitative real-time PCR analysis

Total RNA was isolated from hepatic samples using Trizol reagent according to the manufacturer's protocol. A total of 2.5 *μ*g of RNA was reverse-transcribed into cDNA using SuperScript III First-Strand Synthesis System (Invitrogen, Carlsbad, CA, USA). Quantitative-PCR was performed using the DNA Engine with Chromo 4 Detector (MJ Research, Waltham, MA, USA). The following were added to a final reaction volume of 20 *μ*l: 1x SuperMix (Platinum SYBR Green qPCR Kit; Invitrogen); cDNA (2 *μ*l); and 0.5 *μ*M of each primer. The amplification conditions were as follows: 50 °C (2 min); 95 °C (5 min); followed by 50 cycles of 95 °C (15 s) and 60 °C (30 s).

### Assay of MDA in liver

A part of liver tissue was prepared for homogenization with a buffer containing 0.15 M KCl to obtain 1:10 (w/v) homogenates. The homogenates were then centrifuged at 12 000 × *g* (4 °C) for 20 min to collect the supernatants. The concentrations of MDA activities were measured, as described previously^43^.

### Western blot analyses

Protein was extracted from liver tissue in RIPA buffer together with phosphatase and protease inhibitors. Proteins in SDS-loading buffer were subjected to SDS-12% polyacrylamide gel electrophoresis (PAGE) and transferred to a PVDF membrane (Bio-Rad, Hercules, CA, USA). Antibodies against LC3B, Atg7, Atg5, Beclin-1, p62, *β*-actin (Cell Signaling Technology Inc., Santa Cruz, CA, USA) and PPAR*α* (Abcam, Cambridge, MA, USA), were used for western blot analysis. The membranes were probed with primary antibodies (1:500–1000) in 10 ml of blocking buffer overnight at 4 °C. After washing, the membranes were further probed with the appropriate horseradish peroxidase-conjugated secondary antibody (1:2000) in 10 ml of blocking buffer for 1 h at room temperature. The Supersignal West Pico chemiluminescent substrates (Thermo Fisher Scientific, Rockford, IL, USA) were used for chemiluminescence development. Finally, the quantitative results of western blots were be assessed by ImageJ to make comparisons between different groups.

### Atg7 or PPAR*α* small interference RNA treatment *in vivo*

Autophagy was inhibited through the siRNA against Atg7 (3 mg/kg; Jima, Suzhou); its sequence is 5′-GCAUCAUCUUCGAAGUGAATT-3′. PPAR*α* was inhibited through the siRNA against PPAR*α* (3 mg/kg; Jima, Suzhou); its sequence is 5′-GAGAUCGGCCUGGCCUUCUAAACAU-3′. Atg7 or PPAR*α* knockdown was achieved by siRNA using an Entranster *in vivo* transfection reagent (Engreen Biosystem Co, Beijing) via hydrodynamic tail vein injection in mice. Scrambled siRNA (3 mg/kg) was used as a control. The processes were performed following the manufacturer's instructions.

### Isolation of primary mouse hepatocytes

The mouse livers were perfused with collagenase-containing Hanks' solution at 7 weeks of age, and viable hepatocytes were isolated by Percoll isodensity centrifugation as described.^44^

### Starvation-induced autophagy *in vitro*

Starvation is the most extensively studied condition that induces autophagy. Transfected GFP-LC3 plasmid for 12 h, the primary hepatocytes were subjected to amino acid starvation by incubating for different hours with Earle's balanced salt solution. The percentage of cells with GFP-LC3 puncta were counted in the different groups receiving different treatment. GFP-positive cells were defined as cells that display bright, punctate staining. Approximately 50 cells were counted, and the experiment was repeated at least three times.

### TUNEL assay

Apoptosis in liver sections was detected by TUNEL (red fluorescence) using the *In Situ* Cell Death Detection Kit (Roche, Indianapolis, IN, USA). Negative control was prepared through omission of terminal transferase. Positive controls were generated by treatment with DNase. Nuclei were stained with 49,6-diamino-2-phenylindole (DAPI, 1 *μ*g/ml) for 10 min. Images were performed on a Nikon Eclipse E800 fluorescent microscope (Nikon Corp., Tokyo, Japan).

### Human specimens

Normal liver tissues (*n*=10) were collected from four subjects undergoing hepatic resection for hepatic cyst, and three subjects undergoing hepatic resection for colorectal metastasis, three subjects undergoing hepatic resection for benign tumor, such as angeioma. CHB samples were obtained from the livers of 14 patients undergoing liver puncture biopsy. ALF samples were obtained from the livers of 19 patients with HBV infection undergoing liver transplantation. This study meets the ethical guidelines of the 1975 Declaration of Helsinki, and the study protocol was approved by the Medical Ethics Committee of Beijing YouAn Hospital. Informed consent was obtained from all patients. The clinical characteristics and details of the patients included in the study are shown in Table 1.

### Statistical analyses

The results are shown as the mean±S.E.M. The statistical analyses were performed using an unpaired Student's *t*-test, and *P*<0.05 (two tailed) was considered significant.

## Figures and Tables

**Table 1:** General clinical characteristics of the different study groups

	*Normal subjects (*n=*10)*	*Chronic hepatitis B patients (*n=*14)*	*Acute liver failure patients (*n=*19)*	P-*value*
Age (years)	39.4±3.6	32.4±4.1	41.6±5.3	0.11
Gender (male/female)	6/2	7/5	8/4	0.437
Alanine aminotransferase (U/l)	35.1±6.1	93.3±18.3	210.4±76.3	0.026
Aspartate aminotransferase (U/l)	30.6±3.9	62.6±15.4	305.8±44.6	0.038
Serum bilirubin (*μ*mol/l)	8.8±2.9	20.9±6.3	196.1±50.6	0.01
Prothrombin time (s)	9±2.4	15±5.2	34.4±7.2	0.022
Albumin (g/l)	46.2±6.9	32.6±10.8	26.7±5.7	0.039
Creatinin (*μ*mol/l)	73.6±21.5	80.1±29.0	95.4±32.8	0.042
Hepatic encephalopathy score	—	—	1.7±0.3	—
Child-Pugh score	—	6±0.6	13.7±2.4	0.031
Model for end-stage liver disease score	—	—	28.2±4.2	—
HBsAg test	—	Positive	Positive	—

## Abbreviations

ALF: acute liver failure
ALT: alanine aminotransferase
AST: aspartate aminotransferase
Atg: autophagy gene
CCL: chemokine (C-C motif) ligand
CXCL: chemokine (C-X-C motif) ligand
CHB: chronic hepatitis B
CQ: chloroquine
d-GalN: d-galactosamine
HBV: hepatitis B virus
IL-1*β*: interleukin-1*β*
IL-6: interleukin-6
GSK3*β*: glycogen synthase kinase 3*β*
LPS: lipopolysaccharide
PPAR*α*: peroxisome proliferator-activated receptor *α*
qRT-PCR: quantitative reverse transcription PCR
siRNA: small interfering RNA
3-MA: 3-methyladenine
TNF-*α*: tumor necrosis factor-*α*
TUNEL: terminal deoxynucleotidyl transferasemediated dUTP nick-end labeling
