# Supplementary material for: Inhibition of glycogen synthase kinase 3β promotes autophagy to protect mice from acute liver failure mediated by peroxisome proliferator-activated receptor α
Source: Cell Death Dis. 2016 Mar 24;7(3):e2151–. doi: 10.1038/cddis.2016.56 (PMC4823957; doi:10.1038/cddis.2016.56)
Supplement: Supplementary Figure Legend [file cddis201656x1.docx]

**Supplementary figure legend**

**Supplementary figure 1** Mice were intraperitoneally injected with D-GalN (700 mg/kg) and LPS (10 μg/kg) at 2-, 4-, 6- and 8-hours (10 mice/group). The mice in the control group (n = 8) were injected with PBS only.

**A**. Gene expressions of Beclin-1, Atg5 and Atg7 were measured by qRT-PCR in the livers.

**B**. Protein expression levels of LC3B, p62, Beclin-1, Atg5, Atg7 were measured by western blot assays in the livers. A representative blot from two samples of every group is shown.

**Supplementary figure 2** SB+siRNA PPARα+D-GalN/LPS-treated mice were pretreated with PPARα siRNA (3mg/kg) for 48 hours *via* tail vein injection and then administered SB216763 2 hours prior to D-GalN/LPS exposure (n = 12); SB+siRNA control+D-GalN/LPS-treated mice were pretreated with control siRNA (3mg/kg) for 48 hours *via* tail vein injection, then administered SB216763 2 hours prior to D-GalN/LPS exposure (n = 10);

**A**. Representative livers and H&E staining of livers (400×) from different groups.

**B.** Serum AST and ALT enzyme levels from different groups.

**C**. Gene expression of cytokines including TNF-α, IL-1β and IL-6 at 6 hours, IL-12p40 and IL-10 at 2 hours after D-GalN/LPS injection. Gene expression of chemokines including CCL-1, CCL-2, CXCL-1 and CXCL-10 at 6 hours after D-GalN/LPS injection.
