# Supplementary figures and images for: Inhibition of glycogen synthase kinase 3β promotes autophagy to protect mice from acute liver failure mediated by peroxisome proliferator-activated receptor α
Source: Cell Death Dis. 2016 Mar 24;7(3):e2151–. doi: 10.1038/cddis.2016.56 (PMC4823957; doi:10.1038/cddis.2016.56)

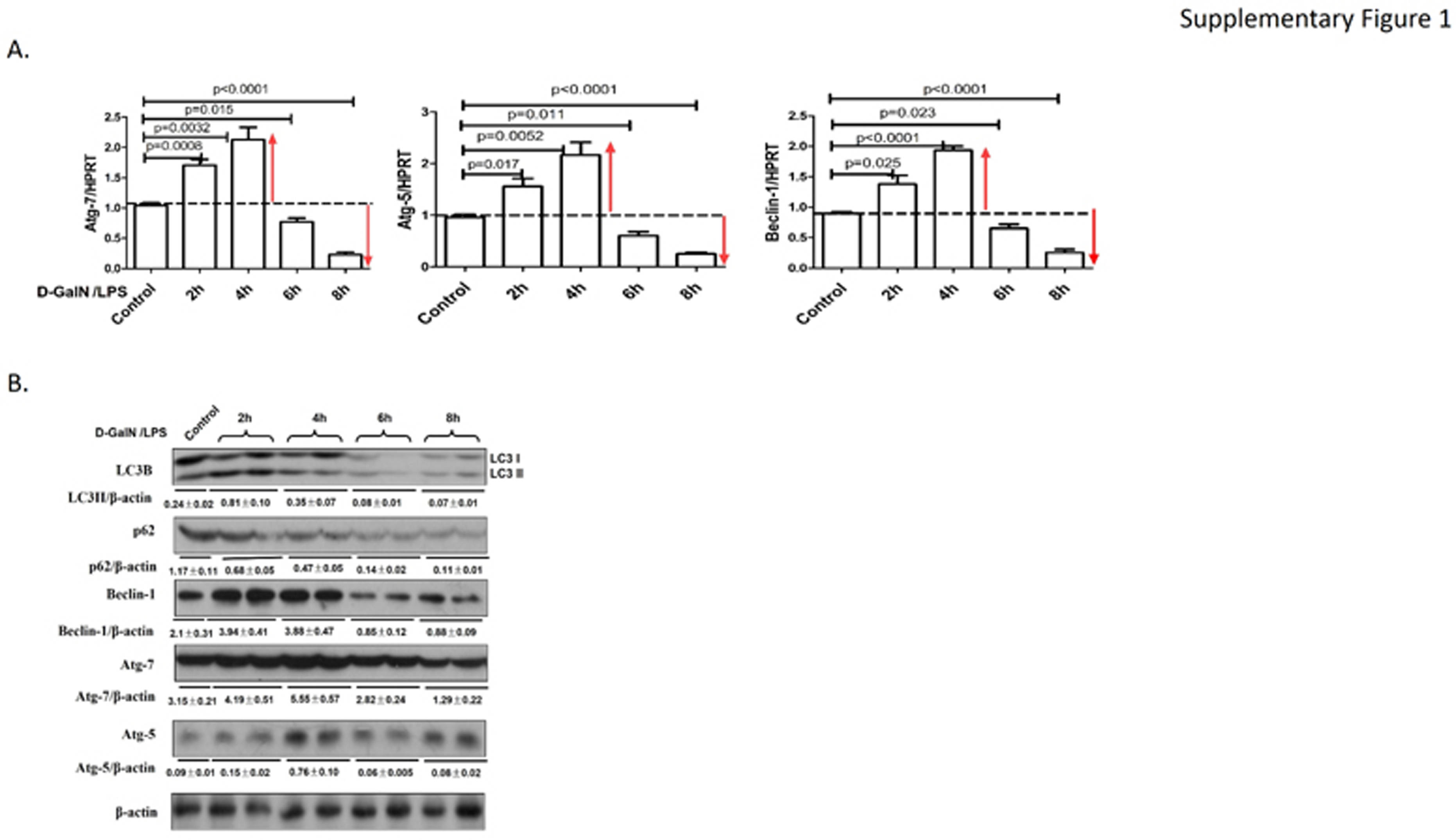

Supplement: Supplementary Figure 1 [file cddis201656x2.tif]

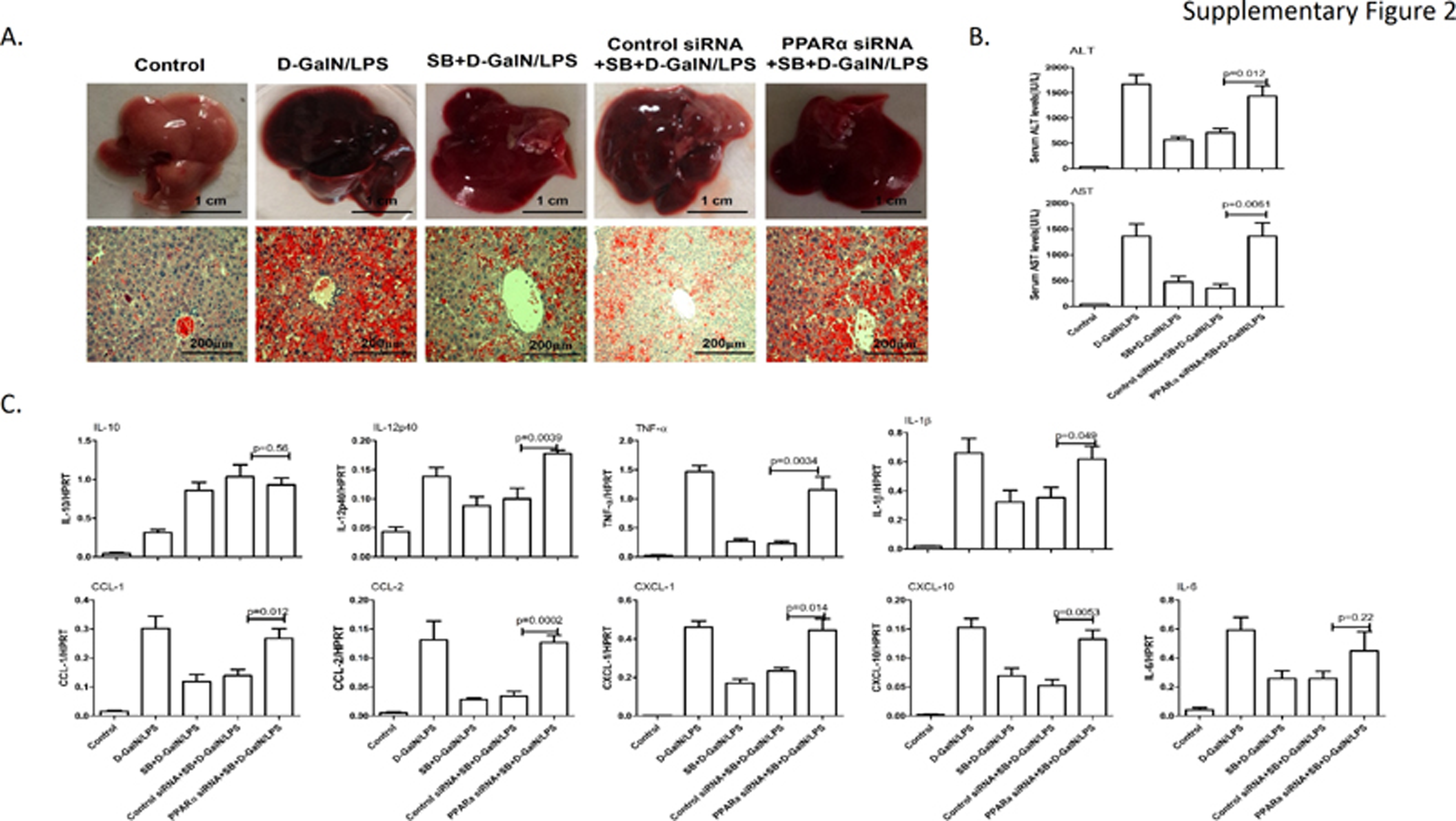

Supplement: Supplementary Figure 2 [file cddis201656x3.tif]
